# Independent living, emotional well-being, and quality of life in people with disabilities: the mediator role of self-determination and satisfaction with participation

**DOI:** 10.3389/fpsyg.2023.1279014

**Published:** 2023-12-21

**Authors:** Blanca Cegarra, Gabriele Cattaneo, Alina Ribes, Javier Solana-Sánchez, Joan Saurí

**Affiliations:** ^1^Institut Guttmann, Institut Universitari de Neurorehabilitació adscrit a la UAB, Barcelona, Spain; ^2^Departament de Teoria i Història de l’Educació, Facultat d’Educació, Universitat de Barcelona, Barcelona, Spain; ^3^Fundació Institut d’Investigació en Ciències de la Salut Germans Trias i Pujol, Barcelona, Spain; ^4^Departament de Medicina, Universitat Autònoma de Barcelona, Bellaterra, Spain

**Keywords:** participation, independent living, emotional well-being, quality of life, disability, self-determination

## Abstract

**Background:**

In the field of disability and rehabilitation, *participation in society* has become an important therapeutic objective due to its potential impact on rehabilitation, prognosis, and patient’s mid and long-term well-being. However, only a few studies have explored this issue in relation with the subjective perspective of individuals with disabilities about their decision-making capacity and satisfaction with the activities in which they participate.

**Objective:**

Our aim is to analyze the relationship between *participation* in society of people with disabilities and both emotional well-being and quality of life, including variables about subjective perspective of *participation* (satisfaction) and the ability to decide and pursue their own preferences.

**Method:**

The cross-sectional study presented here includes 424 participants with disabilities over 18-years-old from Spain. This research is part of a larger ongoing longitudinal cohort study called *Participa* (https://participa.guttmann.com/en/). Participants were asked to complete several on-line surveys to explore quality of life, emotional well-being, *participation* in society, self-determination, and independent living.

**Results:**

The results revealed an association between the dimensions of participation satisfaction, self-determination, and levels of independent living with both quality of life and emotional well-being. Mediation models indicated that satisfaction and self-determination partially mediated the relationship between independent living and both quality of life and emotional well-being.

**Conclusion:**

The level of independent living, self-determination, and satisfaction with *participation* are fundamental aspects for promoting a high quality of life and emotional well-being in people with disabilities. These findings carry significant implications for health and social services, as well as policies guidelines, highlighting the need to prioritize empowerment and self-determination in all interventions designed for people with disabilities.

## Introduction

The study of people’s *participation* in society, how to promote it, and its potential benefits has become increasingly relevant in recent decades. In the field of disability, health and rehabilitation, *participation* has become a therapeutic objective due to its expected impact not only on the rehabilitation process and prognosis, but also on a patient’s mid and long-term well-being.

The International Classification of Functioning, Disability and Health (ICF; WHO, 1980) presents *participation* as one of the fundamental components of its biopsychosocial model. ICF defines it as “the involvement in life situations,” however, as some authors point out, this definition may be too simplistic (Magasi et al., 2018). Moreover, there is still a lack of consensus in the literature on more complex definitions of *participation*, its conceptualization and how to measure it (Whiteneck and Dijkers, 2009; Van De Velde et al., 2010; Hästbacka et al., 2016). Thus, as it is a complex multidimensional concept, there exist a plurality of approaches to define it (Hammell, 2015).

More specifically in relation to *participation*, the ICF definition has been criticized for offering only a limited insight into the type and level of involvement in person’s life situation. Mostly, it can be said that the ICF addresses *participation* through objective performance indicators but does not consider the individual’s subjective perception of what, where, when, how and with whom the person wants to participate.

Furthermore, the study of *participation* should go beyond physical performance and basic activities of daily living, and include broader subjective aspects of life, such as quality of functioning, acceptance, satisfaction, decision-making and control of life or self-determination. These aspects are largely not considered in the ICF definition (see, e.g., Hammell, 2015).

Complementarily, the Independent Living (IL) paradigm proposes that the presence of environmental barriers critically affects the level of independence of people with disabilities and their *participation* in society (DeJong, 1979). This approach provides an alternative analysis of disability problems and their solutions from a social and contextual perspective, going beyond the traditional individual and biomedical perspective. It emphasizes that the problems of people with disabilities are not only physical or biological but are also caused by an unnecessary dependence on family members, caregivers, and healthcare professionals. This dependency should be overcome with new solutions, such as the personal assistance service and accessible housing for fighting forced institutionalization and isolation, and through services oriented to promote independent living and societal *participation*. Here the problem does not lie only with individuals, but also with the environment which restricts the opportunities of people with disabilities to participate in society on an equal basis with others. Therefore, the proposed solutions in these cases are not medical treatments, but rather an increased focus on self-determination, peer support and the elimination of any barrier in the environment.

### Participation in the society, well-being, and quality of life

The study of the relation between dimensions of participation and people’s quality of life and well-being has received more attention in the last decades, revealing a consistent relation between these aspects. Several studies conducted in more than 60 different community provided evidence that increased *participation* of people with disabilities is associated with improved well-being, satisfaction and overall quality of life (Levasseur et al., 2004). For example, it has been showed that greater engagement in valued activities 2 years post stroke was significantly associated with subsequent improvement in emotional well-being (Egan et al., 2014). Similarly, other studies showed that enabling older adults with disabilities to participate in society in its broadest sense can have a positive impact on well-being (van Hees et al., 2020).

However, the relation between *participation* and well-being is not always consistent if subjective indicators of *participation* are not considered. Results of a previous study rejected the hypothesis that higher *participation* by persons with physical disabilities is associated with higher levels of well-being (Van Campen and Iedema, 2007). However, this study only considered objective *participation*, eventually concluding that the subjective aspects of *participation*, such as satisfaction, might have a greater contribution to a person’s well-being.

It has been shown that in European countries, persons with disabilities are in a disadvantaged position in terms of subjective well-being (SWB) (van Campen and van Santvoort, 2013) and it has been suggested that inequality in SWB is explained mostly by personal resources and not by the level of disability, socio-economic status, or level of *participation* in work. Again, also in this case only *participation* with objective indicators, such as the level of *participation* in work, were analyzed.

For years now, the literature has evidenced the importance of subjective indicators of social *participation* to evaluate well-being (Cantor and Sanderson, 1999) and, following this line, Anaby et al. (2011) concluded that satisfaction with *participation* is a more important determinant of well-being than the performance of activities. Thus, the importance of being satisfied with *participation* (deciding what and how the person wants to participate), rather than doing a lot of activities, is evident.

Indeed, Yeung and Towers (2014) showed that quality of life (QoL) in Australian young adults with disabilities is affected by their *participation*, their social relations, and their environment, including connectedness and life satisfaction. They found that satisfaction with *participation* influenced the level of QoL in young adults with disabilities in either a direct or indirect manner. In the case of older people with disabilities, Yeung and Breheny (2021) concluded that purpose in life may help a person to deal with early onset stressors or changes in mental and physical health, therefore maintaining a higher overall quality of life. A study conducted in Canada also found that for older adults with physical disabilities, satisfaction with the accomplishment of life habits is more associated with quality of life than the performance of activities itself (Levasseur et al., 2004).

In the same line also studies conducted in Europe, concretely in Switzerland, indicated that satisfactory *participation* represents a crucial resource for individuals living less than 10 years with a severe spinal cord injury, since it represents buffering potential for the negative effects of chronic pain on mental health and quality of life (Müller et al., 2017).

In this context, our aim is to analyze the relationship between objective and subjective perspective of *participation* (satisfaction), the ability to decide and pursue their own preferences, and their association with emotional well-being and quality of life in people with disabilities.

## Materials and methods

### Participants

424 participants (207 women) from the *Participa* cohort study took part in this study (Cegarra Dueñas and Saurí Ruiz, 2021). Inclusion criteria was to be over 18 years old, have some disability at the time of recruitment, and completed all the questionnaires (no missing data are present in the current analysis).

*Participa*^1^ (Cegarra Dueñas and Saurí Ruiz, 2021) is an ongoing prospective longitudinal cohort study that started in 2020 with the aim to study the barriers and facilitators to *participation* of persons with disability. *Participa* is a social research project aimed at understanding how the *participation* in society of people with disabilities can be guaranteed and promoted, with the intention of reaching fully inclusivity.

*Participa* study volunteers are people with disabilities at the time of recruitment that were recruited by a dissemination and communication campaign made through different media outlets (TV, radio, newspapers), mailing to our database, as well as common social media platforms (Facebook, Twitter, etc.).

People interested in the study enrolled online through the website^2^ by filling out a dedicated form. They gave online informed consent to the study. After verifying email address, participants created their personal profiles and completed an initial online questionnaire. Participants who were minors and those residing outside of Spain were excluded from the study. Participants provided explicit informed consent, and the protocol was approved by the Ethics and Clinical Research Committee of the Institut Guttmann (Spanish neurorehabilitation hospital). The data included in this study were recollected between the beginning of the project and September 2022.

### Procedures

Following the initial questionnaire, participants were asked to complete several additional on-line surveys to further explore quality of life, emotional well-being, *participation* in society, self-determination, and independent living.

#### Initial questionnaire

The first questionnaire, *ad hoc* created for this study, collects socio-demographic and socio-economic data on the participants, such as gender, age, place of residence, income, employment status, type of housing, among other aspects.

#### Participation in society

The Utrecht Scale for Evaluation of Rehabilitation-Participation (USER-Participation) is an ICF-based *participation* measurement instrument that fulfills the need for a brief instrument that contains both objective and subjective *participation* measures. This questionnaire, with a total of 32 items, measures the frequency of participation, experienced participation restrictions, and satisfaction with participation (Van Der Zee et al., 2014). All three scales have a score range of 0–100, with higher scores reflecting better *participation* (higher frequency, less restrictions, higher satisfaction). The internal consistency of this scale, measured by Cronbach’s alphas resulted: 0.68 (C.I.:0.63–0.72) for frequency, 0.78 (C.I.: 0.75–0.81) for restrictions and 0.85 (C.I.:0.83–0.87) for satisfaction.

#### Self-determination

Self-Determination has been measured by the subscale of the GENCAT scale. The construction and validation of the GENCAT scale was based on the multidimensional model proposed by Schalock and Verdugo (2002). In this way, the scale provides valid and reliable scores for eight dimensions, including ‘Self-determination’. Only this subscale has been administered to participants included in this study. Total score ranges from 0 to 36, with higher score reflecting a higher self-determination level. Cronbach’s alpha of this instrument resulted 0.78 (C.I.: 0.75–0.81) in the sample analyzed in this study.

#### Independent living

An *ad hoc* questionnaire has been designed to measure the Independent Living level, based on typical proxies commonly gathered for this concept. The instrument collects information about: (a) The opportunity to choose a place of residence and where and with whom they live on an equal basis with others; (b) The access to a range of in-home and community support services to -support living- and inclusion -in the community, to prevent isolation or segregation from the community; (c) Accessible housing (adequate to the needs of the person); and (d) Affordable housing (less than 40% of income). It consists of 4 items Likert scale ranging from “Totally agree” (4) to “Totally disagree” (0). Total score ranges from 0 to 16, with a higher score reflecting higher levels of independence. The *ad-hoc* created items are reported in the Supplementary material. The reliability coefficient (Chronbach’s alpha) of this scale in our sample is 0.71 (C.I.: 0.67–0.76).

#### Emotional well-being

Emotional well-being was measured with the Spanish version of the ultra-brief self-reported Patient Health Questionnaire (PHQ-4; Kroenke et al., 2009). It consists of 4 items that measure depression and anxiety using a Likert scale ranging from “nearly every day” (3) to “not at all” (0). Total score ranges from 0 to 12, with a higher score reflecting reduced emotional well-being. The reliability coefficient of this scale in our sample is alpha = 0.88 (C.I.: 0.86–0.90).

#### Quality of life

Quality of life has been measured through the first item of WHOQOL-BREF (Spanish version), which asks about self-perceived quality of life, ranging from “very bad” (1) to “very good” (5) (Lucas-Carrasco, 2012).

### Data and statistical analysis

First, we explored the relationship between variables related to *participation* (frequency, restriction, and satisfaction), self-determination and level of independent living running spearman correlations.

Then we explore their association with emotional well-being and quality of life running two multiple regressions, correcting for age and biological sex to prevent bias in the analysis, and checking for collinearity, autocorrelation, and homoscedasticity.

Then, for the variables that showed significant association with emotional well-being and quality of life, we ran mediator’s models using the PROCESS tool for SPSS [model 6; (Hayes, 2012)].

## Results

### Socio-demographic, socio-economic, and disability characteristics of participants

Demographic, socio-economic and disability characteristic of subjects are reported in Tables 1, 2. Participants were younger than general population with disabilities in Spain (2020, data from Statistical Institute of Spain),^3^ where 75.4% of people with disabilities or limitations residing in households are 55 years old or older, and three out of every five persons are women. Because women have a longer life expectancy, there is a larger female population with disabilities in Spain. Thus, women are under-represented in our study. In terms of education, people with superior studies are over-represented (almost 50% compared with 39.7).

**Table 1:** Demographics, socio-economic, and disability characteristics of participants.

Variable	Mean (SD)/Range	Percentage (%)
Age	52.92 (12.52)/ 19–78	-
Sex		Female 48.8
Education level		No studies: 1.2
		Primary: 15.5
		Secondary: 33.5
		Superiors: 49.8
Living with*		Alone: 18.6
		Partner: 51.4
		Fathers: 14.4
		Other family members: 10.8
		Friends: 0.9
		Institution: 0.0
		Other: 6.6
Household Crowding Index (HCI)	0.94 (0.47) / 0.2–2.5	
Working situation		Yes: 20.3
Subsidy		Yes: 76.7
Family incomes (per month)		<500€: 3.5
		From 500€ to 1,000€: 7.3
		From 1,001€ to 1,500€: 12.0
		From 1,501€ to 2,000€: 12.0
		From 2,001€ to 2,500€: 14.9
		From 2,501€ to 3,000€: 16.5
		From 3,001€ to 3,500€: 11.8
		From 3,501€ to 4,000€: 8.7
		>4,000€: 13.3
Severity of disability		33–65%: 31.1
		66–75%: 31.6
		76–100%: 37.3

*The sum of percentages for all items is greater than 100% considering that participants were allowed to choose more than one response in order to better describe their situation (e.g., living with parents and partner at the same time).

**Table 2:** Participants by type of disability.

Variable	Number of subjects
Physical motor disability	373
Non-motor physical disability	90
Visual disability	35
Deafness	24
Intellectual or developmental disability	39
Mental Health problems	39

Only 18.6% of participants reported to live alone, while 76.6% reported to live with partner, fathers, or other family members. Participants, on average, showed a low Household Crowding Index (HCI), being 0.94 of mean. This was calculated by dividing the number of family members by the number of rooms in the house (> 2 is considered crowding). The HCI is related to the socioeconomic status (SES) of the family unit, with 50% of participants having family incomes over 2,500 euros per month.

The working situation of the cohort show that 20% are employed, similar than other data about population with disabilities in Spain. According to the Statistical Institute of Spain, the employment rate for people with disabilities in 2020 was 26.7% (64.3% for people without disabilities).

### Participation, self-determination, and independent living

**Figure 1:**
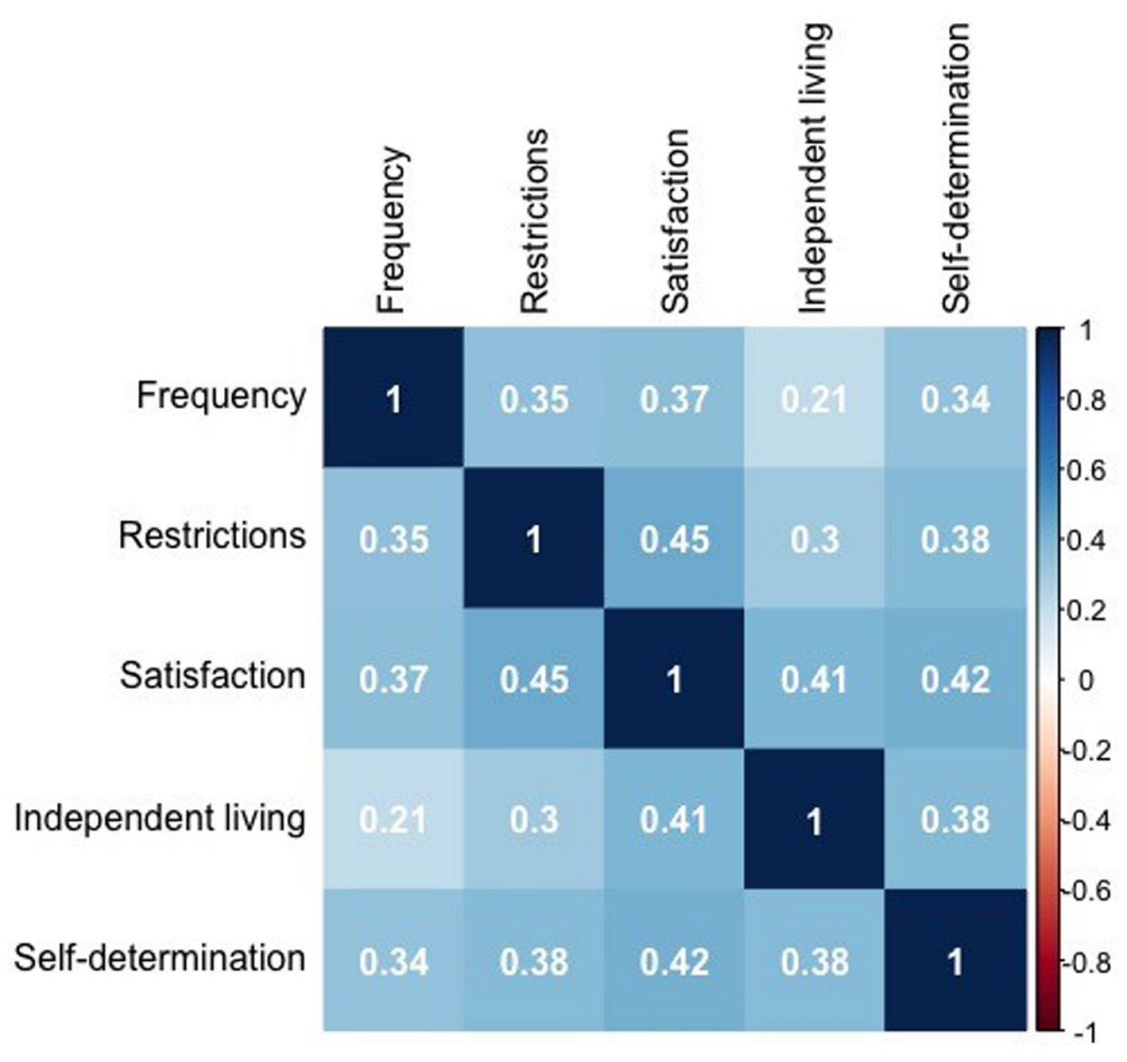
Correlogram representing Spearman’s correlation between variables related with participation, independent living, and self-determination. Numbers in the figure represent the correlation coefficients. Stronger correlations are graphically represented by more intense colors. A legend of colors and correlation coefficients could be find in the y axes on the right side of the figure.

We ran Sperman’s correlation to observe the relationship between variable related with *participation*, self-determination and independent living. All variables resulted moderately associated (See Figure 1; all *p* value <0.001).

### Participation, self-determination, independent living, emotional well-being, and quality of life

Table 3 reports levels of *participation*, self-determination, independent living, emotional well-being and quality of life.

**Table 3:** Participation, self-determination, independent living, emotional well-being, and quality of life reported by participants.

Variable	Mean (SD)/Range
Frequency of participation (USER-P)	31.1 (12.2)/2.9–100
Restrictions of participation (USER-P)	69.2 (18.9)/0–100
Satisfaction of participation (USER-P)	60.8 (19.0)/0–100
Self-determination (GENCAT)	30.6 (4.4)/15–36
Independent living	10.0 (4.1)/0–16
Emotional well-being (PHQ_4)	3.7 (3.1)/0–12
Quality of life (WHOQOL-BREF)	3.0 (1.0)/1–5

### The relation between participation, self-determination and independent living with emotional well-being, and quality of life

#### Emotional well-being

Multiple linear regression showed that age resulted associated with higher emotional well-being (*β* = −0.117, *p* = 0.009), as well as satisfaction with participation (*β* = −0.326, *p* < 0.001), self-determination (*β* = −0.145, *p* = 0.004) and levels of independent living (*β* = −0.119, *p* = 0.018). On the other hand, biological sex (women) was associated with reduced emotional well-being (*β* = 0.089, *p* = 0.040).

**Figure 2:**
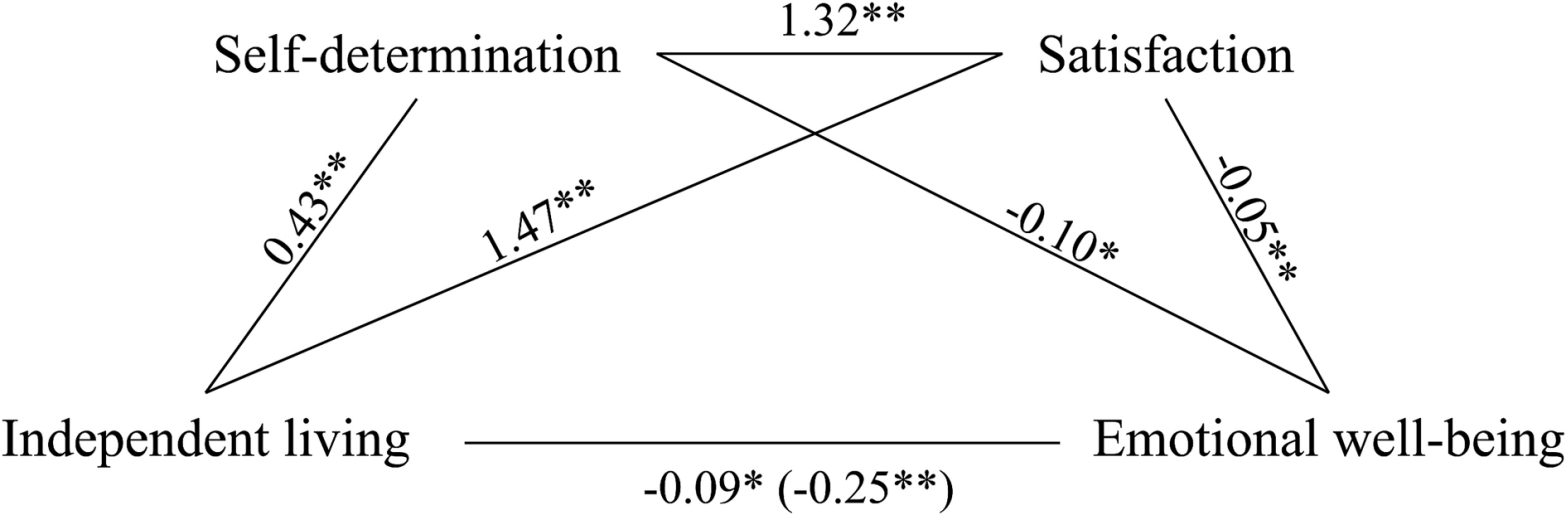
Self-determination and satisfaction as partial mediators of the association between independent life and emotional well-being. Values are B coefficients (*p < 0.05; **p < 0.01); values within parentheses represent total relationship. Path 1: Independent living→Self-determination→Emotional well-being (C.I. 95%: −0.055 to −0.006). Path 2: Independent living→Satisfaction→Emotional well-being (C.I. 95%: −0.162 to −0.069). Path 3: Independent living→Self-determination→Satisfaction→Emotional well-being (C.I. 95%: −0.031 to −0.004).

Bootstrapped mediation analysis revealed that satisfaction and self-determination significantly and partially mediated the association between levels of independent living and emotional well-being (5,000 bootstrap samples, C.I. 95%, see Figure 2). The model explained 13% of the variance without mediators and 26% when mediators were included.

#### Quality of life

Results from the multiple linear regression indicates that age was negatively associated (*β* = −0.150, *p* = 0.001) with quality of life, while satisfaction (*β* = 0.273, *p* < 0.001), self-determination (*β* = 0.178, *p* < 0.001) and levels of independent living (*β* = 0.215, *p* < 0.001) were positively associated to it.

**Figure 3:**
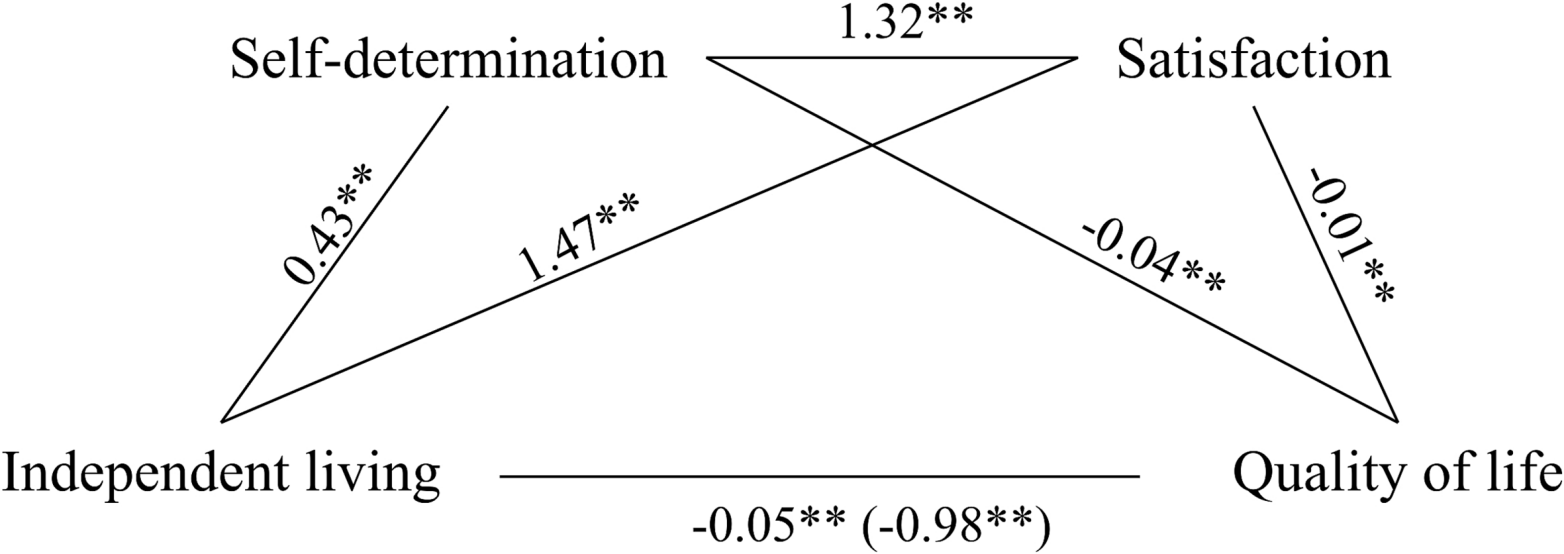
Self-determination and satisfaction as partial mediators of the association between independent life and quality of life. Values are B coefficients (*p < 0.05; **p < 0.01); values within parentheses represent total relationship. Path 1: Independent living→Self-determination→Quality of life (C.I. 95%: 0.007–0.027). Path 2: Independent living→Satisfaction→Quality of life (C.I. 95%: 0.011–0.033). Path 3: Independent living→Self-determination→Satisfaction→Quality of life (C.I. 95%: 0.004–0.013).

Again, in this case, bootstrapped mediation analysis revealed that satisfaction and self-determination significantly and partially mediated the association between levels of independent living and quality of life (5,000 bootstrap samples, C.I.95%, see Figure 3). The model explained 16% of the variance without mediators and 26% when mediators were included.

## Discussion

In this study we aimed to analyze the relationship between *participation* in society, including variables about subjective perspective of *participation* (satisfaction) and the ability to decide and pursue their own preferences, and well-being, in terms of emotional well-being and quality of life of people with disabilities.

We found that levels of *participation* in terms of frequency among individuals with disabilities were low, but paradoxically perception of restrictions was also low, while satisfaction with *participation* was reasonably high. On the other hand, participants reported high levels of self-determination and independent living, and they also reported mild emotional well-being and moderate level of Quality of life. When we explored the relation between these variables, results revealed positive moderate associations between all objective and subjective perspective of *participation* (satisfaction) and the ability to decide and pursue own preferences.

Also, these variables resulted associated with both, quality of life and emotional well-being, and their relationship with independent living and resulted mediated by satisfaction with *participation* and self-determination levels. This underlines the importance of subjective perspective of *participation* to analyze quality of life and well-being of people with disabilities, and the importance of engaging in meaningful activities to perceive good satisfaction with *participation*, good quality of life and emotional well-being.

These results have implications for the health and social services, demonstrating the need for work on empowerment and self-determination in all interventions orientated to people with disabilities (both public and private services). Currently, in Spain, these services (e.g., rehabilitation, personal carers, housing support, subsidies) often take a paternalistic role, instead of promoting maximal levels of independent living and societal *participation* based on individuals’ meaningful activities.

Although Spanish regulations recognize the right to independent living and personal autonomy, public policies do not make these rights effective. For example, Spanish regulations recognize the right to personal assistance (personal support for independent living) for people with high dependency, but in practice, less than 1% of the dependent population makes use of this service (Díaz Velázquez, 2017).

We found that the higher the level of independent living, the higher the levels of self-determination and the greater the satisfaction with *participation*. Consistent with other studies (Mol et al., 2021) participants report in average low frequency of participation but high satisfaction with their participation, and low perception of restrictions. In our study, mean scores on the USER-Participation (Frequency score = 31.1, Restrictions score = 69.2, and Satisfaction score = 60.8) were slightly lower if compared with previous studies conducted in Netherlands and Switzerland in people with Spinal Cord Injury (de Ruijter et al., 2018).

Considering that higher scores reflect more favorable *participation* (higher frequency, fewer restrictions, and higher satisfaction), our Spanish population would be in a worse *participation*’s situation than the other studied countries, may be due to the lack of effectiveness of public policies.

Interestingly, results indicated that lower perception of restrictions were associated with higher levels of *participation*, in line with previous studies (Whiteneck and Dijkers, 2009; Badia et al., 2011; Bezyak et al., 2020) suggesting that the presence of barriers or lack of facilitators reduce activity and *participation*. This is in contrast with the so called “paradox of barriers,” according to which people with higher levels of *participation* are those who report more barriers in their environment (Tsai et al., 2017) due, in part, to the fact that people with disabilities who participate more are those who identify barriers with greater frequency and severity, since they can directly experience and be aware of them (Whiteneck and Dijkers, 2009; Noreau and Boschen, 2010).

This difference could be explained by other aspects that were not considered in our and other studies. Indeed, as suggested by Badia et al. (2011), *participation* is not only related with the perception of barriers, but psychological factors play a role in, possibly modulating this association, and explaining why not always there is a linear relationship between actual barriers, abilities (and other aspects of disability), reported barriers, and *participation* (Dijkers et al., 2002). Future studies must deeper explore the relation between psychological personal profiles and the different variables related with *participation*, in order to better understand how they can impact on the perception of environmental barriers and the awareness about restrictions.

Respect the relation between social *participation* and well-being we found that independent living has a positive impact on the perception of Quality of Life and Emotional well-being and this effect is not totally direct, but partially mediated by other indexes such as self-determination, independent living and satisfaction with *participation*. This is in line with what proposed by Yeung and Towers (2014) about the relationship between quality of life (QoL) in young adults with disabilities and their *participation*, social relations, connectedness and life satisfaction. Moreover, our results support what proposed by Anaby et al. (2011), that beyond physical performance and basic activities of daily living, the analysis of *participation* must include broader subjective aspects of life, such as quality of functioning, self-determination, satisfaction, and consider environmental barriers that could limit people’s levels of independence.

With our study we can assume that being satisfied with *participation* in society is more important in terms of well-being and quality of life than the number of performed activities. It brings greater emotional well-being to perform meaningful activities than to have a very active life without the capacity to make decisions about the performed activities. In relation to that, future studies should address the relevance of different spaces and activities related with *participation* (inclusive or segregated spaces), and their relation to quality of life and emotional well-being.

It is important to analyze “spaces” in which the person participates, given that segregated spaces or itineraries may be barrier’s free, but not necessarily inclusive. For example, leisure and educational spaces designed for people with disabilities could not necessarily appropriate for an inclusive and accessible society.

## Limitations

A primary limitation of this study is that the project employs a fully online enrollment and data collection methodology. This approach may introduce participation bias related to age and other socio-demographic characteristics of participants. As a consequence, the studied sample may not be entirely representative of the Spanish population in terms of age (younger than population), biological sex (under-representation of women) and educational status (higher than population with disabilities in Spain). This must be considered in the interpretation of the results that, however, are in line with previous literature.

Moreover, in this study we did not stratify the analysis by type, severity of disability and gender, due to sample size characteristics. These variables may modulate current results, and future research must investigate the role of them in participation, and its relationship with quality of life and emotional well-being.

## Conclusion

To conclude, our results could have important implications in the rehabilitation field, offering new targets for interventions that focus on promoting empowerment and self-determination, and orienting rehabilitation toward *participation* based on meaningful activities for the patients. All this could have an important impact on the whole rehabilitation process, its prognosis, and finally on patient’s mid and long-term well-being. At the same time, our results evidence the importance of including people with disabilities in the process of designing health and social services and public policies aimed at improving their quality of life. It is important to place the individual at the center of social and health care and to work from the model of person-centered planning.

## Data availability statement

The raw data supporting the conclusions of this article will be made available by the authors, without undue reservation.

## Ethics statement

The studies involving humans were approved by Comitè Ètic d’Investigació (CEIm) de la Fundació Unió Catalana d’Hospitals. The studies were conducted in accordance with the local legislation and institutional requirements. The participants provided their written informed consent to participate in this study.

## Author contributions

BC: Conceptualization, Investigation, Methodology, Writing – original draft. GC: Data curation, Formal analysis, Investigation, Methodology, Writing – original draft. AR: Investigation, Writing – review & editing. JS-S: Conceptualization, Investigation, Supervision, Writing – review & editing. JS: Investigation, Supervision, Writing – review & editing.

## Footnotes

^1^ https://participa.guttmann.com/en/

^2^ https://participa.guttmann.com/en/register-participa/

^3^ www.ine.es
