# Supplementary material for: Independent living, emotional well-being, and quality of life in people with disabilities: the mediator role of self-determination and satisfaction with participation
Source: Front Psychol. 2023 Dec 21;14:1279014. doi: 10.3389/fpsyg.2023.1279014 (PMC10768016; doi:10.3389/fpsyg.2023.1279014)
Supplement: Supplementary file 1 [file Table_1.DOCX]

**Supplementary Material**

1. Spanish version of the ad-hoc created “Independent living scale” employed in the study

| **Spanish Version** |
| --- |
| *Tengo la oportunidad de elegir dónde y con quién vivir* |
| *En el lugar en el que vivo tengo acceso a servicios domiciliarios y comunitarios que me permiten estar incluido/a y participar en la sociedad* |
| *Vivo en una vivienda accesible (adecuada a mis necesidades)* |
| *Vivo en una vivienda asequible (el gasto en vivienda es inferior al 40% de mis ingresos)* |

1. English translation of the ad-hoc created “Independent living scale” employed in the study

| **English Version** |
| --- |
| *I have the opportunity to choose where and with whom to live* |
| *Where I live, I have access to home and community services that allow me to be included and participate in society* |
| *I live in accessible housing (suitable for my needs)* |
| *I live in affordable housing (housing spending is less than 40% of my income)* |
